# Physicians’ experiences of the process leading to their sick leave for exhaustion disorder in Sweden: a narrative design

**DOI:** 10.1136/bmjopen-2024-097179

**Published:** 2025-05-24

**Authors:** Bodil J Landstad, Marit Kvangarsnes, Emelie Thunqvist, Björn Löfman, Åke Nygren, Emma Brulin

**Affiliations:** 1 Faculty of Health Sciences, Mid Sweden University, Östersund, Sweden; 2 Unit of Research, Östersund Hospital, Östersund, Sweden; 3 Department of Health Sciences, Norwegian University of Science and Technology, Trondheim, Norway; 4 Møre og Romsdal Hospital Trust, Alesund, Norway; 5 Department of Occupational Medicine, Karolinska Institutet, Stockholm, Sweden; 6 Center for Occupational and Environmental Medicine, Stockholm County Council, Stockholm, Sweden; 7 Department of Clinical Sciences, Karolinska Institutet, Stockholm, Sweden; 8 Institute of Environmental Medicine, Karolinska Institute, Stockholm, Sweden

**Keywords:** Health Services, Burnout, Professional, Physicians, Nurses

## Abstract

**Abstract:**

**Objectives:**

The aim of this study was to explore physicians' experiences of the process leading up to their sick leave due to exhaustion disorder (ED).

**Design:**

A qualitative study with a narrative approach was conducted.

**Setting:**

The study was conducted in Sweden. Interviews with physicians were conducted face-to-face during the Spring and Autumn of 2022. Each interview lasted between 35 and 60 min.

**Participants:**

Purposive sampling was employed to recruit physicians on long-term sick leave for ED. We used an interview guide with open-ended questions.

**Results:**

12 physicians shared their experiences of the process leading to their ED-related sick leave. Four themes related to different phases of the process: Theme 1. Strongly motivated to become a physician, Theme 2. Demands delivering best practice, Theme 3. Symptoms of ill health and Theme 4. Managing symptoms of ED. The narrative analyses showed a holistic understanding of the personal consequences for the physicians and their families as well as the consequences in working life.

**Conclusions:**

Politicians and leaders at different healthcare levels must take responsibility for strengthening the working environment, which is important for creating long-term and sustainable healthcare services.

**STRENGTHS AND LIMITATIONS OF THIS STUDY**

- The information power in the interviews was strong in relation to the (a) aim of the study, (b) sample specificity, (c) use of established theory, (d) quality of dialogue and (e) analysis strategy.
- The study provided new insights into physicians’ experiences of the process leading to their sick leave due to exhaustion disorder.
- The researchers hold different academic positions in social science, health science, medicine, occupational medicine and psychology, contributing to a comprehensive understanding of the phenomenon.
- The study was solely conducted in a Swedish context, but the findings may be relevant in countries with similar health services.
- Although interviewing only one man might be considered a limitation, it contributes to gender diversity.

## Introduction

 Long-term exposure to stressful work environments is common among physicians and is a well-known challenge in working life.^1^ The risk of sick leave for stress-related ill health is highest in the healthcare sector.^2^ One of the most common consequences of long-term exposure to stress is burnout. Schaufeli *et al.*^3^ conceptualise burnout as a work-related state of exhaustion that leads to a reluctance or inability to expend energy. This results in exhaustion and impaired emotional and cognitive functions, which reduce the ability to manage work tasks or relationships with others. Mental distancing becomes a coping mechanism, which involves psychologically detaching from work as a coping mechanism for exhaustion.^4^,^6^ This can, in turn, intensify stress, leading to further impairment,^3^ eventually becoming persistent and manifesting with severe symptoms.^7^ A recent epidemiological study among working Swedish physicians and nurses showed that nearly 5% showed severe symptoms of burnout.^8^

In Sweden, the term exhaustion disorder (ED) is commonly used for more severe burnout complaints that are clinically assessed by a medical doctor. ED is an established diagnostic term and is included in the Swedish version of the International Classifications of Diseases, 10th edition (ICD‐10 code F43.8).^9^ This means that individuals may be on medically certified sick leave due to ED. According to the diagnostic criteria, ED is marked by extreme fatigue, emotional instability, disrupted sleep and cognitive impairment, including difficulties with attention and executive functions.^9^ Cognitive impairment is a key diagnostic criterion in ED (F43.8) and, therefore, shares many commonalities with the burnout definition proposed by Schaufeli *et al*.^3^ ED often leads to a long period of sick leave due to extensive residual symptoms that affect individual functioning,^9 10^ long-lasting persisting symptoms^9^,^11^ and a high likelihood of recurrence.^12^ Cognitive impairment can persist long after recovery,^10^ impacting work ability. In the following, the term burnout will be used to describe prominent stress symptoms while the physicians are still working. ED will be used to describe the clinically assessed diagnosis.

A large share of quantitative research has established that a poor psychosocial work environment is a risk factor for stress and burnout for physicians.^13 14^ Physicians report feeling powerless to change their situation, facing generic work requirements, such as administration, workload, negative professional culture, complexity of work and occupation-specific work requirements, such as high patient expectations.^15^ In analyses of open-ended survey questions, the physicians described the psychosocial pressures they faced, such as the expectation to handle emotionally distressing events without discussing them and the fear of being judged by senior colleagues for voicing safety concerns.^16^

A few qualitative studies have explored different aspects of physicians' experiences and perceptions of work stress and burnout manifestations. Findings from a study among US primary care physicians identified internal manifestations of stress, expressed as experiencing internal conflicts, moral dilemmas and demoralisation following the sense that work was endless and had a high administrative burden.^17^

Burnout may affect physicians' emotions and behaviours in their professional and personal lives.^13 15 16^ Burnout is stigmatised among physicians, preventing them from seeking help^18 19^ and potentially leading to symptom development. If left untreated, it may advance to sickness absence^20^ and suicide.^20 21^ A systematic review and meta-analysis from 2019 calculated an overall standardised mortality rate of 1.44 for suicides among physicians.^22^ A recent Swedish study showed that suicide was more common among physicians than in the general population.^23^ This calls for a broader understanding of the process leading to burnout and ED among physicians.

A more in-depth understanding of how physicians experience ED is essential for developing knowledge to strengthen the work environment and prevent ED. The study aimed to explore physicians' experiences of the process leading up to their sick leave for ED.

## Methods

We used a qualitative design^24^ with a narrative approach.^24^,^26^ A narrative can be based on stories from one or more persons.^25^ In this study, we chose to conduct narrative interviews with 12 participants. The purposive sampling was based on physicians' unique experiences and insights into the process leading to their ED.

A narrative typically consists of a series of events.^27^ Using a narrative approach gives physicians a clear voice and recognises their significance in the process leading up to ED. By emphasising the physicians' own perceptions of the process, healthcare organisations can gain new insights into their situation and improve their work environment. Narrative analysis follows a theoretical approach that captures personal and human experiences over time while considering the connection between individual experiences and cultural context.^28^ It offers an alternative to viewing individuals as separate from their context, instead recognising them as an integral part of it.^29^

### Data and study population

Physicians on long-term sick leave for ED were identified in a Swedish national insurance database (the Avtalsgruppsjukförsäkringen (AGS) database). The insured physicians who submitted a claim report with ICD codes F32, F41 and F43 in 2021 were informed about the study via email and asked if AGS insurance may disclose contact details to the researchers. 12 participants from all over Sweden were asked and were willing to be interviewed during the Spring and Autumn of 2022. There was no relationship established prior to study commencement. The participants are described in table 1.

**Table 1:** Demographic data on the study participants

	Total n=12
Gender	
Female	11
Male	1
Age	
31–40	2
41–50	6
51–60	4
Place of assignment	
Hospital	11
Primary care	1
Specialisation	
Physician (before residency)	1
Resident	4
Specialist	4
Senior specialist	3
Years of experience	
<5	1
5–10	4
11–15	1
>16	6

### Interviews

The interviews were between 35 min and 1 hour long. They were kept short as the physicians had difficulty concentrating due to their ED. All the interviews were carried out by the last author. Seven interviews were done face-to-face in a specially booked office room at a hotel, and five were done via Teams. Field notes were made during the interviews. All interviews were audiotaped and transcribed verbatim.^24^

We used narrative questions when interviewing the physicians (see box 1).

Box 1 **Narrative questions**

Questions

- Can you tell about your decision to become a physician?
- How did you experience the working life as a physician?
- How did you experience balancing family life and working life?
- What does the work as a physician mean to you?
- How did you experience the working situation before being exhausted?
- What are your thoughts about the future?

### Patient and public involvement

None.

### Data analysis

When analysing the data, we focused on the narrative plot.^30^ First, we gained a holistic impression of the interviews several times.^25^ Meaningful units were identified and coded in relation to different phases before being diagnosed with ED. Using a narrative approach,^30^ focusing on content, form and context in the storyline of the interviews, themes were identified for each phase, and the story was subsequently organised into a chronological structure.^24^

Themes were created by organising the data into increasingly more abstract units. When analysing, we worked back and forth between the data and the themes until we had a comprehensive understanding of the stories.^31^ The research group’s different professional backgrounds were important when interpreting relevant themes. Discussions between researchers were significant in developing an understanding of the narrative itself.^32^ In table 2, we present the analysis process for Theme 4.

**Table 2:** Analysis process for one theme

Data	Subtheme	Main theme
One way to manage the exhaustion was to voluntarily reduce working hours.	Reducing working hours	
“I have realised that I can never go back to my old clinic. The inflow of patients is so large that it’s about to break us all.”	Change of specialty	Managing the ED
Resigning their position and instead working in private practice or at a staffing agency was not foreign to the doctors. They could then work less and have a say in when they want to work.	Private practice or staffing agency	
“I may never be able to return to the medical profession—when I realised that, it felt like life was over.”	Leaving the medical profession	

ED, exhaustion disorder.

## Results

12 physicians shared their experiences of the process leading to their ED-related sick leave. Four themes illustrate the narrative of ED. Theme 1. *Strongly motivated to become a physician*, Theme 2. *Demands delivering best practice*, Theme 3. *Symptoms of ill health* and Theme 4. *Managing symptoms of* ED.

### Theme 1: strongly motivated to become a physician

#### Desire to help

The physicians conveyed their aspiration to help others, expressing a profound sense of fulfilment when they can make a difference in the lives of the ill and vulnerable. They used emotionally charged language, indicating a passion for their profession or a passion for their work. Many had family members who were physicians, and some knew from an early age that they wanted to become physicians. They demonstrated a genuine interest and motivation for their profession.

#### Fulfilment of duties

The physicians reported a significant need to perform and be acknowledged for their accomplishments. One expressed that she needed confirmation of her own significance (P6). This contributed to her difficulty in setting boundaries in her professional life. “It’s hard for me to find balance in my workday. It just doesn’t work. I tend to push on, and then I’m totally exhausted…” (P6).

One specifically expressed that “The job has become so existentially important to me that my entire self-esteem depends on it. When I was on sick leave for the first time due to exhaustion, I thought that my life would essentially cease, and no one would want to be with me anymore and that I would lose everything” (P11). The role of a physician was perceived as a very important part of their identity and self-image.

### Theme 2: demands delivering best practice

#### Engaged and dedicated

Physicians experienced high expectations for their work performance and felt there was no room to be unwell. They shared stories of colleagues who had become acutely ill during work time but continued to work. “There is something that drives us—it is tough to just ignore doing things that can have serious consequences for the patients” (P1). Prioritising their own health was perceived as challenging.

Physicians expressed that they were expected to be self-sufficient after completing their basic medical education (5.5 years of medical school) but had experienced a lack of supervision and follow-up. As junior physicians without a licence or residents, they often received tasks that required specialised knowledge and varied experience. “When my day off finally came, I was extremely tired. The next day, I said to my boss: I have never worked with this, and I need to see how someone else performs the task” (P1). Physicians expressed that they were given a lot of responsibility that they were not ready to take on.

The physicians felt that organisationally, conditions were not set up for them to be able to take care of patient treatment in a qualitatively good way. They experienced the lack of long-term planning in healthcare as imminent. Stories were told about the high turnover of managers, and the work became temporarily planned. The physicians said that they were expected to be very productive and make quick decisions—also difficult decisions—without enough time and resources to make thorough assessments. This, in combination with a substantial administrative burden, an increased number of reports and increased pressure for everything to be documented correctly, had made the workload feel unbearable.

Hired foreign physicians could be perceived as an increased burden on permanent staff because they did not have sufficient knowledge of the Swedish healthcare context. The physicians experienced that they spent a lot of time explaining and guiding them through administrative rules and system understanding.

Of the physicians interviewed, several had tried to ‘put their foot down’ and protested what they perceived as working conditions that negatively affected patient treatment. The physicians experienced that their employer did not understand their dedication to providing best practices. They experienced it as burdensome to stand up for quality patient treatment.

The physicians reported a change in recent years. Many patients had high health literacy and were knowledgeable about their disease. This was perceived as positive, and these patients were motivated to follow treatment advice. The physicians also felt that some patients had unrealistic expectations for treatment and health services.

#### Patient care

Physicians experienced a lot of administration and documentation. If not enough time was given to these tasks between patient visits, it became difficult to switch and be an active and empathetic listener when the next patient arrived. Often, they had to postpone the administrative tasks until after work hours or the next day. This created stress, and doctors expressed that they were afraid of forgetting important things.

The physicians experienced that there was no time for recovery between tasks. Previously, they had staff who could help with scheduling, follow-ups and other administrative tasks. “All this secretarial work that one now does… It’s not sensible” (P3). Physicians did not perceive this as a good use of physicians' resources to do a thorough job.

The physicians emphasised the importance of being focused and caring. Following up on unstable patients could be the difference between life and death. Good communication with patients and relatives was crucial. Physicians reported that when they became exhausted, they had more difficulty listening to, taking the patient’s perspective and meeting the patient’s emotional needs.

#### Work and life balance

Some of the physicians had substantial caregiving responsibilities within their own families. This could be challenging to combine with a high workload and irregular working hours. “One feels so confined when working in healthcare. If I must make any changes, it becomes extremely difficult. Then I must juggle my calendar, and it becomes stressful when I try to participate in meetings with the school or anything else concerning family members” (P1). Choosing between work and family could lead to experiences of ethical dilemmas.

### Theme 3: symptoms of ill health

#### Anxiety and symptoms of depression

The physicians described that before they were diagnosed with ED and on sick leave, they had been pushing their limits for a long time. It could be temporary things that became the triggering factor. It was described in terms of being ‘a bit like pulling the plug on the air mattress’. Many went in and out of short-term sick leave before they finally went on long-term sick leave.

The physicians reported that they had difficulty interpreting their own symptoms. Some had been investigated for a variety of things before it was diagnosed as ED. Others had thought that they had developed anxiety or depression before it was concluded that it was ED. Fatigue, sleep difficulties and cognitive difficulties were not put in context with deficiencies in the work environment and high workload.

#### Cognitive changes

The physicians described that they had been experiencing personal changes over a long period of time. They had become quiet, tired and listless and had withdrawn from the community both at work and at home. Their ability to concentrate had also failed, and it had been challenging to keep their thoughts together. Several had felt anger and had outbursts over small things. The physicians found this difficult and uncomfortable.

There were stories of overwhelming fatigue. “For a while, I tried to go and take a power nap during the non-existent lunch—just a quarter of an hour to try to survive, but no—then I just became more and more tired. I started to lose words, drop things, became clumsy, could not coordinate, and did not understand what I was reading. Then I got an incredible headache—all the time. I vomited in the mornings before I went to work. I kept going like that for a while before I was put on sick leave…” (P3). The physicians experienced that their described cognitive and mental changes were incompatible with ensuring patient safety in care and treatment.

#### Guilt and shame

The physicians blamed themselves for becoming exhausted. They believed they had themselves to blame because they were unable to limit their tasks, had been too meticulous, had not had time to acquire enough knowledge, received enough training, etc. There was an expressed shame towards colleagues because they left them in an even worse situation when they were absent due to sick leave. The physicians often experienced dark thoughts and subsequent low self-image and described a change from mastering their tasks to becoming insecure and having problems making decisions in patient treatment.

The physicians conveyed that they felt burdened with guilt by their immediate boss when they called and told them that they were on long-term sick leave. “What are we going to do with your patients now?” was a recurring question.

The physicians felt that there was great uncertainty among health personnel about how to treat a colleague with ED. They were ignored and could experience a feeling of wanting to shout out “see me—ask those uncomfortable questions—I am not plague infected” (P6). The physicians felt that they became alone and isolated in the work environment.

### Theme 4: managing symptoms of ED

#### Reducing working hours

The physicians reported that one way to manage their symptoms of ED was to reduce working hours voluntarily. Some chose to work half time to be able to follow-up on the children and their activities. One of the physicians expressed that it was not very smart, considering future pensions, but that there and then, it felt like the easiest way to solve a difficult situation.

#### Change of specialty

The physicians looked at the possibility of changing specialties as a way to create a better working environment and better conditions. “I have realised that I can never go back to my old clinic. The inflow of patients is so large that it’s about to break us all” (P1).

Changing specialty would mean several years of further residency, but several physicians reasoned that they would instead take that burden than leave the profession. Some had thought of switching to a specialty where they do not have to be on call duty.

#### Private practice or staffing agency

The physicians were not foreign to the idea of resigning their positions and instead working in private practice or at a staffing agency. They reasoned that they could then work less and have a say when they wanted to work. The idea of living away from the family for periods, however, was not appealing. Some reasoned that this opportunity opened up as soon as the children had moved out.

#### Leaving the medical profession

The physicians had considered leaving the medical profession. One expressed it like this: “I may never be able to return to the medical profession—when I realised that, it felt like life was over” (P5). This description shows how important the medical profession was to the informants and how great a loss it would entail to leave the profession. The physicians showed a strong commitment to continue to provide healthcare to their patients. “I really feel that I can make a difference, and I become stressed and disappointed that the employer does not give me the tools and conditions to do a good job for the patients” (P4). These statements show the physician’s positive commitment and strong engagement in the medical profession.

## Discussion

The physicians’ narratives conveyed their experiences of the process leading to their sick leave for ED. The narrative shows how physicians were strongly motivated to work in the medical profession and dedicated to delivering best practices for patients. Symptoms of ED evolved over time, and they experienced a lack of understanding from management and colleagues. The physicians used different strategies to manage the exhaustion, such as reducing working hours, changing residency, choosing to work in private practice or staffing agency and leaving the medical profession. The process of being burned out, resulting in sick leave for ED, had huge consequences for the physicians' and patients' access to medical services.

The physicians expressed that they were expected to be self-sufficient after completing their basic education (5.5 years of medical school). They were given responsibility for demanding tasks without having previous experience. Lack of supervision and follow-up characterised the work environment. These situations created stress, and they experienced a lack of coping. Earlier research has shown that feelings of stress, frustration or distress are expected to be hidden or tightly controlled by physicians to appear competent, calm, empathetic and confident.^33^ Physicians experiencing ED may be perceived as weak, incompetent and less of a doctor.^18^ In a working environment like this, it is a risk that young physicians are waiting too long to seek help for their shortcomings and ask for supervision. Jain *et al.*^34^ described that residents often prioritise the patient, leading to self-neglecting their health and well-being.

The physicians expressed high professional requirements for ethics and quality in their work. Not being able to provide best practices could contribute to experiences of ethical dilemmas. This is in line with the findings of Agarwal *et al.*,^17^ who showed that primary care physicians experienced professional dissonance, that is, feelings of discomfort arising from the conflict between professional values and job demands.

The administrative burden was expressed as problematic for the interviewed physicians. A review by Patel *et al.*^35^ shows that the administrative burden in healthcare is demanding and can override the quality time physicians need for patient assessment. The administrative burden may lead to exhaustion, depersonalisation, substance abuse and depression.^35^ Swedish studies have shown that administrative burdens surrounding performance-based reimbursement systems are associated with both physician burnout^36^ and quality of care^37^ by causing moral distress.

The narratives showed that choosing between work and family could lead to ethical dilemmas. Not being able to follow-up on the parental role was experienced as problematic. This is in line with previous studies where physicians described neglecting family life or not having the energy to engage in family and social activity.^34^ Research has shown that burnout in female physicians was significantly associated with conflicts in work and life balance,^38^ which leads to burnout and job dissatisfaction.^13^ Thus, there is a risk of creating a vicious circle where work stresses negatively impact private life, which in turn leads to burnout.

An important finding is that the physicians did not feel that their work needs were met. This may be related to deficient knowledge among managers and colleagues about ED and how the work environment should be designed to prevent ED. These findings are consistent with those of Petri *et al.*^33^ among junior medical officers in Australia. It may also be related to the stigma towards burnout,^19 39^ causing managers and colleagues to turn a blind eye to the burned-out physician.

The narrative highlighted the physicians' struggle to work despite having symptoms of ED. Other studies found that physicians with burnout experience losing empathy and apathy towards patient suffering and depersonalisation.^17 34 40 41^ Physician burnout is acknowledged to lead to malpractice and poor quality of care^42^ and impact how they treat patients.^41^ The narrative highlighted the struggle the physicians had working despite having symptoms of ED. Other studies found that physicians with burnout experienced losing empathy and apathy towards patient suffering and depersonalisation.^17 34 40 41^ It is acknowledged that physician burnout may lead to malpractice and poor quality of care^42^ and impact how they treat patients.^41^

The stigma concerning stress and burnout among physicians may prevent them from seeking help for their symptoms. Physicians are less likely than others to seek help for burnout symptoms.^43^ Not seeking help can worsen their health, leading to ED and long-term sick leave with pronounced symptoms^10 11 44^ and potentially suicide.^20 22 23^ Not seeking help also means prolonged suffering with burnout symptoms that need to be handled. The physicians used different strategies to manage the exhaustion, such as reducing working hours, changing residency, choosing to work in private practice or staffing agency and even considering leaving the medical profession. Reduced work effort due to exhaustion may result in a lack of medical services. It is, therefore, important to take proactive measures to prevent physicians from becoming exhausted and dropping out of working life. Every physician leaving the medical profession is a huge cost to society. Leaving the medical profession was also experienced as a great loss for the interviewed physicians.

### Strengths and limitations

Qualitative research offers a powerful tool for in-depth, rich information regarding a phenomenon’s experiences.^24^ This study’s major strength is the rich and thick information given in the stories of lived experiences told by the physicians who were on sick leave for ED. The data provide new knowledge about what contributed to ED among physicians. We considered the information power in the interviews to be strong depending on the (a) aim of the study, (b) sample specificity, (c) use of established theory, (d) quality of dialogue and (e) analysis strategy.^45^ 12 interviews provided rich data to give new insight into physicians’ experiences of the processes leading up to sick leave for ED. In narrative research, the researchers are cocreators of the narrative. The researchers' knowledge can contribute to a more holistic and credible narrative.^27^

Another strength is that the researchers hold different academic positions in social science, health science, medicine, occupational medicine and psychology, contributing to a comprehensive understanding of the phenomenon. Their competencies were important in analysing the interviews and creating the narrative.^25^

Although it might be seen as a weakness that only one man participated in the study, this provides variety in gender. Further research from a gender perspective can provide new and interesting insights.

The study was solely conducted in a Swedish context. Nevertheless, our findings may be relevant in countries with similar health services.^24 25^

## Conclusion

The narrative provides a new understanding of the personal consequences and suffering of the process leading to physicians' ED. The findings showed that physicians wished to be good healthcare providers but that burnout symptoms made it a struggle, potentially affecting patient safety and society. Less administrative burden, less time pressure for documentation, greater support for work-life balance and mechanisms to reduce workloads when needed are necessary to prevent ED. Managers should pay particular attention to early symptoms of ED among physicians. Leaders at different levels of healthcare organisations must learn about ED and its causes and prioritise a healthy and sustainable work environment for physicians. How to prevent ED should be a topic in medical education as well as specialist training for physicians.

## Data Availability

No data are available.
