# Supplementary material for: Physicians’ experiences of the process leading to their sick leave for exhaustion disorder in Sweden: a narrative design
Source: BMJ Open. 2025 May 24;15(5):e097179. doi: 10.1136/bmjopen-2024-097179 (PMC12104901; doi:10.1136/bmjopen-2024-097179)
Supplement: Supplementary file 1 [file bmjopen-2024-097179.reviewer_comments.pdf]

PEER REVIEW HISTORY

BMJ Open publishes all reviews undertaken for accepted manuscripts. Reviewers are asked to complete a checklist review form and are provided with free text boxes to elaborate on their assessment. These free text comments are reproduced below.

ARTICLE DETAILS

Title (Provisional)

Physicians' experiences of the process leading to their sick leave for exhaustion disorder in Sweden: a narrative design

Authors

Landstad, Bodil J.; Kvangarsnes, Marit; Thunqvist, Emelie; Björn, Löfman; Nygren, Åke; Brulin, Emma

---

VERSION 1 - REVIEW

**Reviewer**	**1**
**Name**	**Linzer, M**
**Affiliation**	**Hennepin Healthcare, Medicine**
**Date**	**28-Feb-2025**

COI **I perform burnout reduction studies for large health care systems such as the AMA, IHI, and the Optum Office for Provider Advancement. Support is paid to my employer, Hennepin Healthcare. These would not be significant conflicts for my reviewing the current paper.**

Some very important and sad findings about highly burned-out physicians. The Methods are sound, including the analytic strategy, and the major limitation (to be added to the limitations section) is 12 respondents; 31 consented, and it is not clear how many were asked (would specify if available). So, generalizability is not certain; also as the authors note 11 of the 12 were female, so not likely representative of the general physician group. The quotations are profoundly meaningful (doctors highly burned out reported being told by supervisors, "what are we to do with your patients (if you take time off)?" it is so sad to think of that being said. Leadership training in empathy could be valuable as a suggested outcome.

They also note that going part-time status is effective, which has been reported before but is always worth restating.

The Conclusions could be a bit stronger: what recommendations would the authors offer, such as less administrative burden, less time pressure for documentation, greater support for work-life balance and mechanisms to reduce workloads when needed. it seems they

have gained insights into severe burnout; telling us their ideas on how to address it before it gets to this stage would be appreciated.

I congratulate the authors on this fine work. The findings are distressing, and if remediable and preventable, would be a major contribution.

Reviewer **2**

Name **Moir, Fiona**

Affiliation **The University of Auckland, General Practice and Primary Healthcare**

Date **21-Mar-2025**

COI **None**

This is an under-researched area (the period leading up to taking sick leave), as it's known that many doctors do not take sick leave but continue to work when unwell. Therefore, a study which explores this adds to the literature.

Page 3, Line 6: Regarding the aim, the phrase 'physicians' experiences of the process' seems quite broad to me – what process ? their illness experience, the processes in their workplace?

Page 3, Line 10: 'purposeful sampling' – I suggest changing to 'purposive'. This is an appropriate sampling method for the design.

Page 3, Line 23: Theme 2 'Demands delivering best practice' – can this theme be written more clearly?

Page 4, lines 14-18: I agree with the first point about what this particular study adds to the literature; however, I'm not quite clear about what 'vast consequences' refers to – in the period leading up to the sick leave? (which is what this study is exploring).

Page 5, Line 11: "one of the most common consequences of long-term exposure to stress that is not adequately managed is burnout". This could perhaps be expanded upon of clarified to make it clear about what is meant by 'adequately managed' – are the authors referring to the individual person or to the system?

I felt that these 3 consecutive sentences below were a somewhat repetitive way to describe the primary symptoms of burnout on page 5:

• Lines 11-21 : Schaufeli et al. (3) have conceptualised burnout as "a work-related state of exhaustion that occurs among employees, which is characterised by extreme tiredness, reduced ability to regulate cognitive and emotional processes and mental distancing" .

• Primary symptoms of burnout include a reluctance or inability to expend energy, resulting in exhaustion, impaired emotional and cognitive functions, and mental distancing.

• Lines 18-21: Emotional and cognitive impairments are often seen as the central aspects of burnout because they reduce the ability to manage emotions and cognitive tasks.

Page 5, Line 31: I would suggest putting this excellent paragraph first. Many readers may not be familiar with Exhaustion Disorder as a diagnostic term, so the authors may wish to consider using their initial paragraphs to clarify the differences and similarities between the terms burnout and ED. Is the concept of 'Burnout' in the Swedish equivalent of the ICD labelled 'Exhaustion Disorder' ? What are the similarities and differences? With phrases in the manuscript like "severe symptoms of burnout that would likely be diagnosed as ED if clinically assessed", it would be helpful to have some clarification of this. As a reader I asked myself – is burnout similar to ED? , a subset of ED? is ED a consequence of burnout? etc.. It is very interesting to come across a different term and it would be good to have some clarification.

Page 6, line 40: I would have liked to see more information included about the narrative approach that was taken. The references cited here appear to be complete books, with no specific chapter indicated. The data analysis section on page 8, provides some details, but some more of this information could be summarised in the abstract.

In general, the Results are clearly stated.

Page 17: The strengths of the study are clearly stated, however, the limitations could be more fully outlined.

Page 17: I am not sure that a conclusion can be drawn that managers much pay 'particular attention – in young female physicians'. It just happened that all bar one of the participants were women. But that doesn't mean that this study shows that they are more at risk than men.

I'm afraid I'm not quite clear how all 6 authors meet the ICMJE recommendations for authorship. All authors are stated to have contributed to 'investigation'; however, all of the 12 interviews were conducted by one person. What does investigation encompass? Only one author is stated as having contributed to 'analysis'. It's not clear how all authors have met all 3 of these criteria below:

• Substantial contributions to the conception or design of the work; or the acquisition, analysis, or interpretation of data for the work; AND

• Drafting the work or revising it critically for important intellectual content; AND

• Final approval of the version to be published; AND

There is also one set of author initials (BA) which do not correspond with the author name at the start of the manuscript.

VERSION 1 - AUTHOR RESPONSE

	**Reviewer 1**			
	The Methods are sound, including the analytic strategy, and the major limitation (to be added to the limitations section) is 12 respondents; 31 consented, and it is not clear how many were asked (would specify if available).	This was an inaccurate description of the recruitment. 12 people were asked to be interviewed and all of them agreed to participate.<br><br>In the Strengths and limitations section we have added text about information power of the study.	Methods<br><br>Strengths and limitations	Page 5, line 6-9<br><br>Page 15, line 2-7
	The Conclusions could be a bit stronger: what recommendations would the authors offer, such as less administrative burden, less time pressure for documentation, greater support for work-life balance and mechanisms to reduce workloads when needed.	We have re-written the Conclusion section and are now more specific in our recommendations.	Conclusion	Page 15, line 23-28
	**Reviewer 2**			
	Page 3, Line 6: Regarding the aim, the phrase 'physicians' experiences of the process' seems quite broad to me – what process? their illness experience, the processes in their workplace?	In narrative research, the aim is to give informants a strong voice. Open-ended	Aim	Page 4, line 19-21

		questions were used that invited them to tell and emphasize what they experienced as important - without being directed by the researcher.		
	Page 3, Line 10: ‘purposeful sampling’ – I suggest changing to ‘purposive’. This is an appropriate sampling method for the design.	We have replaced purposeful sampling with purposive sampling.	Methods	Page 4, line 26
	Page 3, Line 23: Theme 2 ‘Demands delivering best practice’ – can this theme be written more clearly?	We believe that the sub-themes clarify the consequences of the theme and therefore choose to keep it.	Methods	Page 8, line 7 and 31
	Page 4, lines 14-18: I agree with the first point about what this particular study adds to the literature; however, I’m not quite clear about what ‘vast consequences’ refers to – in the period leading up to the sick leave? (which is what this study is exploring).	We have deleted the bullet points according to suggestions from Editor.	What this study adds	Page 2
	Page 5, Line 11: “one of the most common consequences of long-term exposure to stress that is not adequately managed is burnout”. This could perhaps be expanded upon of clarified to make it clear about	We agree that this is an unclear sentence and have removed the text “that is not	Introduction	Page 3, line 4-5

	what is meant by 'adequately managed' – are the authors referring to the individual person or to the system?	adequately managed".		
	I felt that these 3 consecutive sentences below were a somewhat repetitive way to describe the primary symptoms of burnout on page 5:<br>• Lines 11-21 : Schaufeli et al. (3) have conceptualised burnout as "a work-related state of exhaustion that occurs among employees, which is characterised by extreme tiredness, reduced ability to regulate cognitive and emotional processes and mental distancing" .<br>Primary symptoms of burnout include a reluctance or inability to expend energy, resulting in exhaustion, impaired emotional and cognitive functions, and mental distancing.<br>• Lines 18-21: Emotional and cognitive impairments are often seen as the central aspects of burnout because they reduce the ability to manage emotions and cognitive tasks.	We have edited the text to be less repetitive. The text now reads "Schaufeli et al. (3) conceptualise burnout as a work-related state of exhaustion that leads to a reluctance or inability to expend energy. This results in exhaustion and impaired emotional and cognitive functions, which reduce the ability to manage work tasks or relationships with others. Mental distancing becomes a coping mechanism which involves psychologically detaching from work as a coping mechanism for	Introduction	Page 3, line 5-10

		exhaustion (4–6). This can, in turn, intensify stress, leading to further impairment (3), eventually becoming persistent and manifesting with severe symptoms (7)."		
	Page 5, Line 31: I would suggest putting this excellent paragraph first. Many readers may not be familiar with Exhaustion Disorder as a diagnostic term, so the authors may wish to consider using their initial paragraphs to clarify the differences and similarities between the terms burnout and ED. Is the concept of 'Burnout' in the Swedish equivalent of the ICD labelled 'Exhaustion Disorder'? What are the similarities and differences? With phrases in the manuscript like "severe symptoms of burnout that would likely be diagnosed as ED if clinically assessed", it would be helpful to have some clarification of this. As a reader I asked myself – is burnout similar to ED?, a subset of ED? is ED a consequence of burnout? etc. It is very interesting to come across a different term and it would be good to have some clarification.	We have rewritten this section to describe ED in relation to burnout better. The ED/burnout aspect is not straightforward as several definitions exist of burnout, i.e., Maslach's definition does not include cognitive impairment, which is a central aspect of ED. However, cognitive impairment is included in the definition of burnout that we include in this study (Schaufeli et	Introduction	Page 3, line 14-26

		al.). Meanwhile, the word exhaustion is used in Sweden to describe burnout complaints that are not clinically assessed. The term exhaustion denotes just one component of burnout as defined by Schaufeli et al. Nevertheless, we hope that we have managed to make it clearer for the reader by adopting the text to common international terminology.		
	Page 6, line 40: I would have liked to see more information included about the narrative approach that was taken. The references cited here appear to be complete books, with no specific chapter indicated.	We have added more text about the narrative approach that was chosen.	Methods	Page 4-5, line 29-2
	The data analysis section on page 8, provides some details, but some more of this information could be summarised in the abstract.	We have added more details from the data analysis section.	Abstract	Page 2, line 5-9

	Page 17: The strengths of the study are clearly stated, however, the limitations could be more fully outlined.	We have added text to give a more holistic picture of the strengths and limitations.	Strengths and limitations	Page 15, line 1-14
	Page 17: I am not sure that a conclusion can be drawn that managers much pay ‘particular attention – in young female physicians’. It just happened that all bar one of the participants were women. But that doesn’t mean that this study shows that they are more at risk than men.	We have deleted the wording “… in young female physicians”. The text now reads: “Managers should pay particular attention to early symptoms of ED among physicians.”	Conclusion	Page 15, line 25
	I’m afraid I’m not quite clear how all 6 authors meet the ICMJE recommendations for authorship. All authors are stated to have contributed to ‘investigation’; however, all of the 12 interviews were conducted by one person. What does investigation encompass? Only one author is stated as having contributed to ‘analysis’. It’s not clear how all authors have met all 3 of these criteria below:<br>• Substantial contributions to the conception or design of the work; or the acquisition, analysis, or interpretation of data for the work; AND<br>• Drafting the work or revising it critically for important intellectual content; AND<br>• Final approval of the version to be published; AND	We have revised and specified the authors contribution.	Contributorship statement	Page 16, line 8-15

	There is also one set of author initials (BA) which do not correspond with the author name at the start of the manuscript.			

VERSION 2 - REVIEW

Reviewer 1
Name Linzer, M
Affiliation Hennepin Healthcare, Medicine
Date 17-Apr-2025
COI

Excellent revisions; all concerns addressed. This is an important, meaningful and also sad presentation about how burnout can be accompanied by devastating consequences (emotional disorder) requiring doctors to take time away from work, suffer mental health consequences and consider leaving the profession. The authors do an excellent job demonstrating several key aspects of this condition, and the quotes are very moving. They also offer specific actionable steps organizations can take to avoid this. I congratulate them on this work.

Reviewer 2
Name Moir, Fiona
Affiliation The University of Auckland, General Practice and Primary Healthcare
Date 14-Apr-2025
COI

All previous comments thoroughly addressed. Congratulations on an excellent paper.
